# Efficiency of Innovative Extraction Technologies in Recovering Natural Pigments, Polyphenols, and Antioxidants from Sugar Beet Leaves

**DOI:** 10.3390/molecules31213948

**Published:** 2026-11-02

**Authors:** Franka Markić, Senada Muratović, Višnja Stulić, Tomislava Vukušić Pavičić, Sanda Pleslić, Nadica Maltar-Strmečki

**Affiliations:** 1 Laboratory for Electron Spin Spectroscopy, Division of Physical Chemistry, Ruđer Bošković Institute, Bijenička c. 54, 10000 Zagreb, Croatia; fmarkic@irb.hr (F.M.); smuratov@irb.hr (S.M.); 2 Laboratory for Food Processes Engineering, Department of Food Engineering, Faculty of Food Technology and Biotechnology, University of Zagreb, Pierottijeva 6, 10000 Zagreb, Croatia; visnja.stulic@pbf.unizg.hr (V.S.); tomislava.vukusic.pavicic@pbf.unizg.hr (T.V.P.); 3 Department of Applied Physics, University of Zagreb Faculty of Electrical Engineering and Computing, Unska 3, 10000 Zagreb, Croatia; sanda.pleslic@fer.unizg.hr

**Keywords:** sugar beet leaves (SBL), by-products, bioactive compounds, antioxidant activity (AA), microwave-assisted extraction (MAE), pulsed electric field (PEF), ultrasound-assisted extraction (UAE), valorisation

## Abstract

The growing demand for agri-food by-product valorisation has driven the adoption of innovative extraction techniques. This study evaluates and compares pulsed electric field (PEF), ultrasound-assisted extraction (UAE), and microwave-assisted extraction (MAE) for the recovery of total polyphenols (TPC), chlorophylls, and antioxidant activity (AA) from sugar beet leaves (SBL). The outcomes depended strongly on the applied technology and the properties of the target compounds. PEF treatment at 7.5 kV for 3 min resulted in a TPC of 11.41 mg GAE/g dry matter, while the highest chlorophyll content (0.393 mg/g dry matter) was obtained at 2 kV for 3 min. UAE using 80% methanol provided 8.23 mg GAE/g dry matter TPC after 5 min, whereas the highest chlorophyll content (0.601 mg/g dry matter) was achieved after 3 min. MAE resulted in the highest TPC (23.75 mg GAE/g dry matter) at 60 °C, 5 min, and a 1:40 solid-to-solvent ratio, while the highest AA (44.57 mg AAE/g dry matter) was obtained at 80 °C, 10 min, and the same 1:40 ratio. Overall, these findings demonstrate that target-specific extraction strategies are essential for SBL valorisation. These results represent the best-performing conditions within the investigated experimental ranges and provide an initial basis for further research into the potential of these technologies for SBL valorisation.

## 1. Introduction

The food industry generates substantial amounts of waste throughout food processing. In the context of a sustainable bioeconomy, increasing attention is being directed towards minimising waste generation by valorising these by-products as valuable food ingredients, additives, or sources of bioactive compounds, thereby enhancing the value of food products while reducing the accumulation of food waste [1,2]. Sugar beet leaves (SBL) represent an abundant but underutilised agricultural by-product with considerable potential as a source of valuable bioactive compounds. Sugar beet production has traditionally focused primarily on the roots, which serve as the main raw material for sugar manufacturing and, to a lesser extent, for applications such as fibre production and fermentation [1]. In contrast, the leaves, which represent a major by-product of sugar beet processing, have attracted increasing interest as a potential source of valuable compounds. Their considerable availability, together with their rich composition of proteins, polyphenols, flavonoids, and other antioxidant compounds, makes SBL a promising raw material for further valorisation [2].

Although SBL show considerable potential for valorisation, the selection of an appropriate extraction and processing technologies is essential to ensure efficient recovery of bioactive compounds. Conventional extraction methods often suffer from drawbacks such as high solvent consumption, long extraction times, and the potential degradation of thermolabile compounds [3,4]. Nevertheless, solvent extraction, maceration, and solid–liquid extraction has been used to recover bioactive compounds from SBL [5,6,7]. To overcome the limitations of conventional methods, several alternative extraction technologies have been investigated. Ultrasound-assisted extraction (UAE), microwave-assisted extraction (MAE), pressurized liquid extraction (PLE), and subcritical water extraction have been evaluated for polyphenol recovery from SBL [6]. Other studies have explored the use of UAE to recover polyphenols, proteins, and other specialized plant metabolites from this by-product [8,9]. In addition, plasma-assisted extraction using high-voltage electrical discharge (HVED) has been investigated for the recovery of bioactive compounds from SBL [10], whereas studies specifically investigating pulsed electric field (PEF) for this purpose appear to be lacking in the available literature. Against this background, the present study evaluates PEF, UAE, and MAE for the recovery of bioactive compounds from SBL [11]. These technologies may enhance mass transfer and reduce processing times, potentially contributing to more efficient and sustainable extraction processes [5,10,12].

However, the recovery of bioactive compounds from SBL remains challenging due to the complex composition of the plant matrix and the presence of diverse bioactive compounds and pigments with different physicochemical properties and stability. Moreover, many of these compounds are sensitive to processing conditions and may undergo degradation or oxidation, potentially affecting the quality, safety, and functional properties of the obtained extracts [1,13]. Therefore, the optimisation of processing conditions is essential to enhance the recovery of target bioactive compounds while preserving their stability and beneficial properties. In this context, a direct comparative evaluation is important to determine which processing parameters have the greatest influence on compound recovery and to identify the most suitable technology for the efficient, targeted extraction of specific groups of bioactive compounds.

In addition to the quantification of bioactive compounds, the evaluation of their antioxidant properties provides important information on the functional quality of the obtained extracts. Conventional methods for assessing antioxidant activity are widely used; however, electron paramagnetic resonance (EPR) spectroscopy represents a highly sensitive and reliable complementary approach for studying free radicals and antioxidant activity [14]. Its high sensitivity and selectivity towards paramagnetic species make EPR a valuable tool for monitoring changes in free radical formation and scavenging during food processing, thereby providing deeper insights into the mechanisms underlying the observed antioxidant effects [1,10,15].

Therefore, the aim of this study was to investigate and compare the efficiency of selected emerging processing technologies, namely PEF, UAE, and MAE, for the recovery of bioactive compounds from SBL. The study evaluated their effects on the recovery of total polyphenols and chlorophylls, as well as the resulting antioxidant activity, and identified the best-performing processing conditions within the investigated experimental ranges. Furthermore, this work aimed to assess the applicability of EPR spectroscopy as an analytical tool for characterising the antioxidant properties of sugar beet leaf extracts obtained under different processing conditions. This initial comparative assessment was intended to explore the potential of these technologies for SBL valorisation and to provide a basis for further research into potential applications of SBL-derived extracts.

## 2. Results

### 2.1. Pulsed Electric Field (PEF) Assisted Extraction

**Figure 1:**
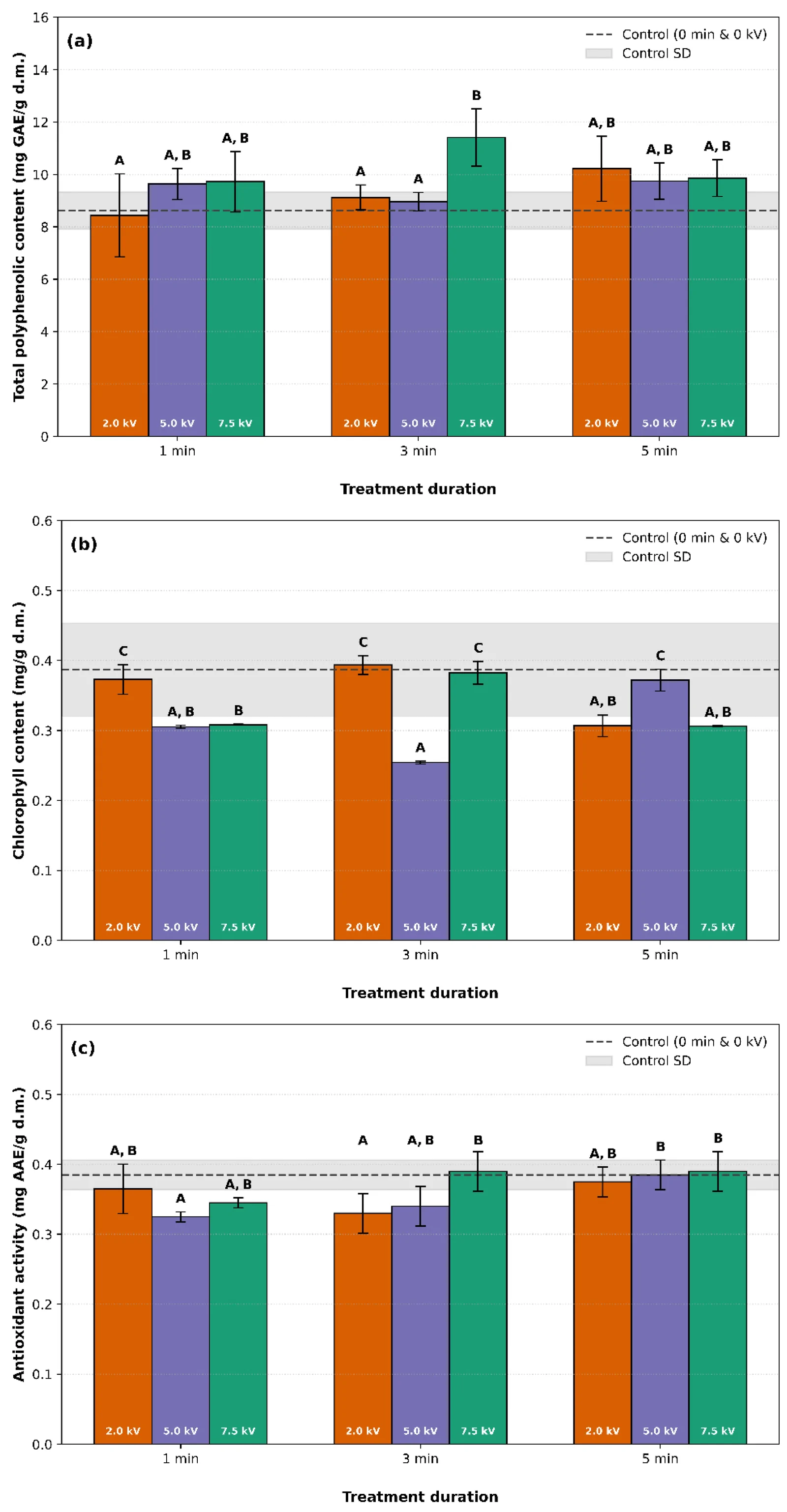
(**a**) Total polyphenolic content (TPC), (**b**) chlorophyll content, and (**c**) antioxidant activity (AA) of SBL extracts under various PEF conditions (treatment duration and applied voltage). The dashed line and shaded area show the control group mean ± SD. Bars not sharing a common letter are significantly different (Fisher’s LSD test, *p* < 0.05). The control group corresponds to statistical group “**A**” in panel (**a**), group “**C**” in panel (**b**), and group “**B**” in panel (**c**).

The total polyphenolic content (TPC), total chlorophyll content, and antioxidant activity (AA) of sugar beet leaves (SBL) extracts obtained under various PEF processing conditions are presented in Figure 1. The obtained results fall within the range reported in previous studies on sugar beet leaves subjected to different extraction approaches [1,2,5,6,16,17]. Previous chromatographic investigations [1] have shown that SBL contain a diverse profile of phenolic compounds, with apigenin dihexoside and hexopentoside, flavonol dihexoside, vitexin, (iso)rhamnetin hexopentoside, acylated apigenin hexopentoside, and diferulic acid among the compounds frequently reported as major constituents.

The PEF dataset was analysed using a unified one-way ANOVA framework encompassing all 10 distinct operational configurations (df*_model_* = 9, df*_error_* = 10), with a single shared untreated control (0 min, 0 kV). Pairwise comparisons were performed using Fisher’s Least Significant Difference (LSD) test at the 95% confidence level.

For TPC, no statistically significant differences were observed among the investigated configurations (*p* = 0.2090), despite numerical variations in the measured values (Table 1). Most PEF-treated samples exhibited higher TPC values than the untreated control ((8.62 ± 0.50) mg GAE/g d.m.). The values ranged from 8.44 mg GAE/g d.m. at 2 kV for 1 min to a maximum of 11.41 mg GAE/g d.m. following treatment at 7.5 kV for 3 min. Although this maximum represented a numerical increase relative to the control, the overall analysis did not confirm statistically significant differences among the treatment configurations.

In contrast, chlorophyll content differed significantly among the investigated PEF configurations (*p* = 0.0017). The lowest value (0.254 mg/g d.m.) was recorded following treatment at 5 kV for 3 min, corresponding to group A. This value differed significantly from those obtained for the untreated control and several other configurations assigned to group C. Treatments at 2 kV for 1 and 3 min, as well as at 7.5 kV for 3 min, showed chlorophyll contents comparable to the untreated control, as indicated by their shared group C designation. The remaining configurations exhibited intermediate values, with their statistical relationships depending on the assigned letter groups.

Regarding AA, no statistically significant differences were detected among the investigated configurations (*p* = 0.1119). The measured values showed numerical variation, ranging from 4.08 ± 0.16 mg AAE/g d.m. at 5 kV for 1 min to 4.89 ± 0.41 mg AAE/g d.m. at 7.5 kV for 5 min. Despite this numerical range, AA did not exhibit a statistically significant response to the applied PEF conditions. Furthermore, the configuration yielding the highest numerical AA value did not correspond to the configuration yielding the highest TPC, indicating that the numerical variations in these two parameters did not follow the same pattern across the investigated treatments.

### 2.2. Ultrasound-Assisted Extraction (UAE)

**Figure 2:**
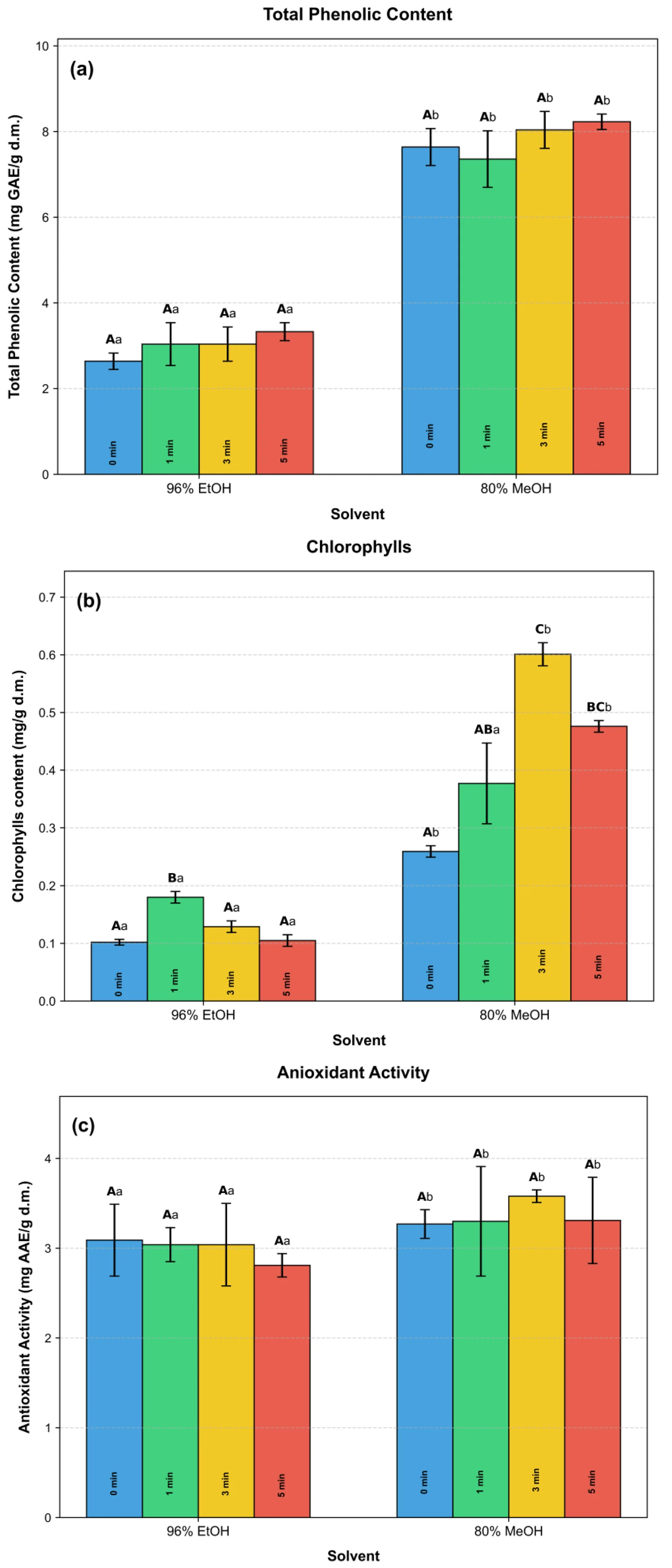
(**a**) Total polyphenolic content (TPC), (**b**) chlorophyll content, and (**c**) antioxidant activity (AA) of SBL extracts under various UAE conditions (treatment duration and solvent type). Different uppercase letters (**A–C**) within columns indicate significant differences between treatment durations; lowercase letters (a, b) indicate differences between solvent type (*p* ≤ 0.05, Tukey post hoc).

The TPC, total chlorophyll content, and AA of SBL extracts obtained via UAE under different treatment times and solvent compositions are summarised in Figure 2. As shown by the ANOVA results (Table 2), both treatment duration and solvent type exerted highly significant effects on chlorophyll extraction (*p* < 0.001). Furthermore, a significant interaction effect (Treatment duration × Solvent) was established for chlorophyll recovery (*p* < 0.001). Conversely, for TPC and AA, solvent type was identified as the sole independent factor exerting a highly significant impact (*p* < 0.001).

The experimental data demonstrated that solvent type was the primary determinant governing the recovery of bioactives during UAE. Methanolic extracts consistently achieved significantly higher TPC, chlorophyll content, and AA than their ethanolic counterparts (*p* ≤ 0.05, Table 2). Within the methanolic extraction series, TPC showed a numerical increase with sonication duration, reaching the highest value of (8.23 ± 0.18) mg GAE/g d.m. after 5 min. However, treatment duration did not have a statistically significant effect on TPC (*p* = 0.153).

Although solvent type significantly affected TPC and AA, numerical differences were also observed across treatment durations. Increasing the sonication time from 1 to 5 min (further details in 4.2.2.), corresponding to an escalation in cumulative energy input from 5.4 to 27.0 kJ (acoustic energy density: 0.216 to 1.080 kJ/mL), followed with an increase in TPC from (7.36 ± 0.66) to (8.23 ± 0.18) mg GAE/g d.m. However, this numerical increase was not statistically significant.

Regarding chlorophyll content, both extraction duration and solvent interactions caused sharp changes, with the highest concentration observed in the methanolic extract after 3 min of sonication (0.601 mg/g d.m.), followed by a reduction after 5 min of treatment (0.476 mg/g d.m.). Similarly, the highest AA value in the methanolic extracts was observed after 3 min (3.58 mg AAE/g d.m.), followed by a numerical decrease after 5 min (3.31 mg AAE/g d.m.). Nevertheless, treatment duration had no statistically significant effect on AA (*p* = 0.820).

### 2.3. Microwave-Assisted Extraction (MAE)

The quantitative distribution of total polyphenolic content (TPC), chlorophyll content, and AA of SBL extracts across the different MAE conditions is summarised in Table 3.

The three-way analysis of variance (ANOVA) demonstrated that MAE parameters—namely temperature, treatment duration, and solid-to-solvent ratio (SSR)—as well as their interactions, exerted highly significant effects on the monitored parameters of SBL extracts (Table 4).

For the TPC, all main effects were statistically significant, with temperature (*F* = 27.869, *p* < 0.001), treatment duration (*F* = 13.739, *p* = 0.003), and SSR (*F* = 19.717, *p* < 0.001) profoundly influencing the extraction yield. Furthermore, the binary interactions of Temperature × SSR (*F* = 36.521, *p* < 0.001) and Treatment duration × SSR (*F* = 28.387, *p* < 0.001) displayed the highest *F*-ratios in the TPC model, while the three-way interaction (Temperature × Treatment duration × SSR) was also significant (*F* = 4.012, *p* = 0.046). As shown in Table 3, TPC values varied widely across treatments, ranging from a distinct minimum of (2.90 ± 1.58) mg GAE/g d.m. (80 °C, 5 min, 1:20 SSR) to a maximum nominal peak of (23.75 ± 2.32) mg GAE/g d.m. obtained under milder thermal conditions (60 °C, 5 min, 1:40 SSR).

Chlorophyll content was exceptionally sensitive to changes in processing conditions, with temperature (*F* = 251.51, *p* < 0.001) and SSR (*F* = 177.81, *p* < 0.001) emerging as the most dominant individual factors. All two-way interactions were statistically significant, including Temperature × Treatment duration (*p* = 0.023), Temperature × SSR (*p* < 0.001), and Treatment duration × SSR (*p* = 0.025), whereas the three-way interaction was non-significant (*p* = 0.788). A clear, downward numerical trend in chlorophyll levels was observed with increasing extraction temperature and higher solvent volumes (SSR). The highest pigment retention was recorded at 60 °C, 10 min, and 1:20 SSR (0.029 ± 0.000) mg/g d.m.), whereas the lowest nominal value dropped to (0.013 ± 0.001) mg/g d.m. at 80 °C, 5 min, and 1:40 SSR.

Statistical analysis showed no significant impact of temperature (*p* = 0.255), treatment duration (*p* = 0.255) and SSR (*p* = 0.095) on AA. However, the three-way interaction (Temperature × Treatment duration × SSR) was statistically significant (*F* = 6.393, *p* = 0.013), as well as combined interaction of Temperature × Treatment duration (*p* = 0.004) and the Treatment duration × SSR interaction approached the significance threshold (*p* = 0.051). The AA values fluctuated drastically, dropping to nearly undetectable levels (<0.01 ± 1.38) mg AAE/g d.m.) at 80 °C, 5 min, and 1:40 SSR, but reaching a substantial peak of (44.57 ± 4.47) mg AAE/g d.m. when the treatment time was extended to 10 min at the same temperature and SSR (80 °C, 10 min, 1:40 SSR).

Some variability among triplicate measurements was observed for certain MAE treatments, particularly for TPC and AA, as reflected by the relatively higher standard deviations obtained for these responses. This variability may be associated with the inherent heterogeneity of the plant material and the sensitivity of the extraction process to small variations in sample composition and experimental conditions. In comparison, chlorophyll measurements showed generally lower variability among triplicates. Nevertheless, the observed variation was taken into account in the statistical analysis and did not prevent the identification of clear differences among the investigated extraction conditions.

### 2.4. The Hierarchical Cluster Analysis (HCA)

**Figure 3:**
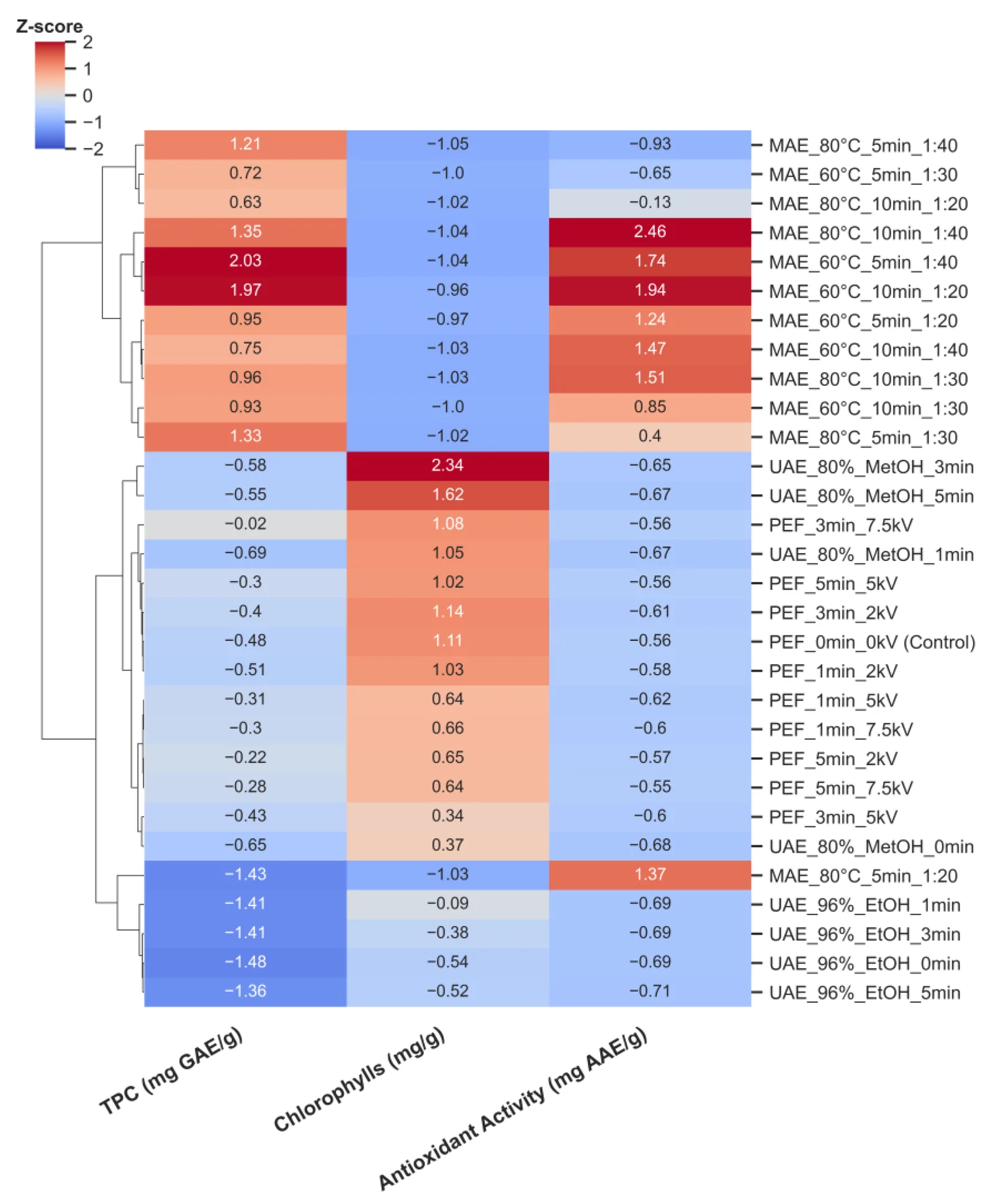
The clustered heatmap displaying the hierarchical cluster analysis (HCA) of SBL extracts obtained across 30 experimental runs utilizing PEF, UAE, and MAE. Z-scores were calculated globally across all investigated extraction techniques (PEF, UAE, MAE) to allow direct multi-method comparison.

The vertical dendrogram (left axis) illustrates the agglomerative grouping of extraction runs computed via Ward’s minimum variance method based on Euclidean distance metrics (Figure 3). The horizontal axis represents the locked, non-clustered sequence of investigated response variables: TPC, Chlorophyll Content, and AA.

The color-coded matrix scale indicates the standardized Z-score values, where deep red segments denote high, above-average component recovery and deep blue segments signify low, below-average values. The numeric values embedded within the cells represent the exact calculated Z-scores for each respective operational condition.

A hierarchical cluster analysis (HSA) performed on 30 experimental conditions successfully identified the natural grouping of samples into three distinct and technology-specific clusters based on the phytochemical and antioxidant profile of the SBL extracts:

Cluster 1: High Polyphenolic Efficiency Cluster (MAE-Dominant)

Microwave-assisted extraction, particularly under 80 °C/10 min/SSR 1:40 conditions, emerged as highly efficient for isolating polyphenols. This cluster is characterized by exceptionally high, above-average TPC (up to 19.64 mg GAE/g) and antioxidant activity (up to 44.57 mg AAE/g) values. Notably, while most MAE runs in this cluster exhibited below-average chlorophyll values, the specific run MAE 80 °C 10 min 1:40 showed a strong positive outlier effect for chlorophyll retention (Z-score of 2.46), indicating that chlorophyll behavior under MAE is highly sensitive to specific operational interactions.

Cluster 2: High Chlorophyll Selectivity Cluster (UAE-MetOH Dominant)

Ultrasound-assisted extraction utilizing 80% methanol forms a separate group defined by the preservation and massive co-extraction of green pigments, reaching its peak at 3 min of treatment (0.601 mg/g).

Cluster 3: Low-Yield and Non-Thermal Screening Cluster (PEF and EtOH-UAE)

This large cluster encompasses the remaining experiments, in which recovery was generally in the range from below average to moderate. PEF across all operational modes (1–5 min, 2–7.5 kV) display high data homogeneity, forming a tight sub-cluster with balanced, close-to-average profiles (stable, moderate to high positive Z-scores for chlorophylls paired with stable, moderate TPC responses). Through non-thermal electroporation, PEF successfully circumvents the degradation risks observed in MAE (where unfavorable conditions such as 80 °C/5 min/1:20 trigger a TPC collapse to 2.90 mg GAE/g). PEF maintains stable and high levels of both chlorophylls (0.38 mg/g) and TPC (11.41 mg GAE/g at 3 min/7.5 kV). In contrast, switching the UAE solvent to 96% EtOH forced those runs into the lowest-performing primary branch of the dendrogram, characterized by a continuous block of deep blue cells across all three response variables. Furthermore, the highly unfavorable thermal run (MAE 80 °C 5 min 1:20) was statistically clustered at the bottom of this group due to its severe TPC collapse (Z-score of −1.43).

MAE emerged as a highly efficient technology for maximizing TPC and AA under specific high-dilution conditions (1:40 SSR). This trend is mathematically reflected by the row-wise dendrogram of the HCA, which isolates the majority of intensive MAE runs into a distinct Cluster 1 (Figure 3). Within this zone, the clustering highlights high TPC and AA responses. Notably, while several MAE runs exhibited below-average chlorophyll values, the optimal run MAE 80 °C 10 min 1:40 stood out as an exception, demonstrating high positive Z-scores across all three investigated variables, including chlorophyll content (2.46).

In contrast, UAE utilizing 80% methanol provided a strong chlorophyll recovery paired with lower-to-moderate TPC and antioxidant activity compared to the top per-forming MAE runs. The HCA algorithm handles this distinct behavior by constructing a separate, highly specialized Cluster 2 for these specific UAE-MetOH runs (Figure 3). Within this mathematical matrix, the sample processed with 80% methanol for 3 min stands out as a unique peak, exhibiting the absolute maximum positive Z-score for chlorophyll content (2.34) across the entire study, while holding negative Z-scores for both TPC and antioxidant activity.

When the solvent is switched to 96% EtOH, the cluster affiliation shifts entirely (cluster 3). The EtOH-UAE runs are forced by the agglomerative algorithm into the lowest-performing primary branch of the dendrogram, characterised by a continuous block of deep blue cells across all three response variables. This mathematical arrangement proves that for the ultrasound-assisted extraction of SBL, the choice of solvent polarity is a more dominant processing factor than the treatment duration. Furthermore, an unfavorable thermal run (MAE 80 °C 5 min 1:20) was also statistically grouped at the bottom of this branch due to its severe TPC collapse (Z-score of −1.43).

PEF exhibited an intermediate, balanced performance for all three response variables. The HCA model groups the entire PEF dataset into an exceptionally compact, homogenous sub-cluster (Figure 3). The remarkably low internal node distance within this branch graphically underscores the high stability and reproducibility of pulsed electric field. In this zone, the mathematical model yields a balanced profile, showing stable, moderate-to-high positive Z-scores for chlorophylls paired with stable, moderate TPC responses.

## 3. Discussion

### 3.1. Effects of Pulsed Electric Field (PEF) Treatment

To date, no prior study has detailed the application of PEF explicitly SBL. However, several studies have evaluated PEF-assisted extraction from red beet matrices, yielding comparable outcomes regarding the influence of treatment intensity. According to Yeasmen et al. (2025), no significant differences in TPC were observed between red beet samples treated with 50 and 90 pulses at a field strength of 2.5 kV/cm [18], indicating that variations in PEF intensity do not necessarily translate into enhanced polyphenol extraction. These findings [18] align with the results obtained in the present study. In contrast, Tekin et al. (2026) observed significantly higher TPC and antioxidant activity in PEF-treated red beetroot [19], with the greatest enhancement achieved at lower energy inputs, whereas further increases in energy resulted in a decline of both parameters. This discrepancy suggests that while optimal PEF conditions can stimulate extraction, an excessive or inadequate energy input may fail to provide incremental benefits or even induce degradation.

Regarding the pigment profile, although no significant differences in chlorophyll content were observed among the investigated PEF treatments in this study, the literature demonstrates that PEF can enhance chlorophyll recovery in certain matrices, particularly microalgae [20,21]. This enhancement has been primarily attributed to PEF-induced cell membrane electroporation, which accelerates mass transfer and facilitates the release of intracellular pigments. Furthermore, the non-thermal nature of PEF and the application of mild processing conditions typically support the preservation of such thermosensitive pigments during extraction [22].

However, the overall efficiency of PEF strongly depends on the structural and textural characteristics of the processed material, which explains why similar improvements have not been consistently reported for leafy plant tissues. Taken together, the absence of statistically significant differences between the PEF-treated samples and the untreated control indicates that the investigated treatment conditions did not produce a statistically significant improvement in the measured responses. Further investigations should examine a broader range of PEF parameters to better understand their influence on extraction efficiency in the SBL matrix. Since PEF-assisted extraction relies on electroporation to facilitate the release of intracellular compounds, future studies could explore different electric field strengths and treatment durations to determine whether alternative processing conditions improve the recovery of the target bioactive compounds.

### 3.2. Effects of Ultrasound-Assisted Extraction (UAE)

The results demonstrate that solvent chemistry was the dominant factor governing the recovery of bioactive components during UAE. The significantly higher yields of TPC, chlorophylls, and AA in 80% methanol compared to 96% ethanol highlight the importance of solvent polarity and affinity in cavitation-mediated extraction. Although solvent type significantly affected both TPC and AA, the difference between the solvents was more pronounced for TPC than for AA. This suggests that a higher polyphenol content does not necessarily translate into a proportional increase in antioxidant activity, as AA may also be influenced by other bioactive compounds present in SBL that were not investigated in the present study but may have substantially contributed to the measured AA values.

Nevertheless, the acoustic energy delivered during UAE also modified the extraction dynamics, particularly within the methanolic medium. Extending the sonication period from 1 to 5 min increased the total energy input from 5.4 to 27.0 kJ, corresponding to an acoustic power density (APD) increase from 0.216 to 1.080 kJ/mL. This enhanced acoustic energy input was accompanied by a progressive elevation in polyphenol release, as evidenced by the TPC rising from 7.36 mg GAE/g d.m. (1 min) to 8.23 mg GAE/g d.m. (5 min). A similar positive correlation between ultrasound exposure and TPC yields from sugar beet leaves was previously documented by Nutter et al. (2021) [17]. Although numerical variations in TPC and AA were observed with increasing sonication duration, these trends were not statistically significant. Therefore, prolonged sonication cannot be considered to have significantly improved either response under the investigated conditions. In contrast, solvent type significantly affected both TPC and AA, highlighting the importance of solvent composition when interpreting UAE results.

A comparable relationship regarding radical scavenging activity was observed by Maravić et al. (2022) [6], where ultrasound-assisted processes significantly enhanced AA relative to conventional solid–liquid extraction methods, an effect directly ascribed to the accelerated cavitation-induced recovery of free phenolic fractions and associated redox-active constituents. However, in the current study, the AA within the methanolic extracts reached its experimental maximum after 3 min of sonication (3.58 mg AAE/g d.m.), indicating that excessive energy input or prolonged acoustic cavitation beyond this threshold might induce localised thermal or oxidative degradation of specific highly reactive antioxidant structures [23,24]. The observed effects may be related to ultrasound-induced acoustic cavitation, where the formation and collapse of bubbles generate localised shear forces and microstreaming, enhancing solvent penetration and facilitating the release of intracellular compounds [25].

The effect of ultrasound energy input on chlorophyll recovery followed a different pattern, with the highest chlorophyll concentration (0.601 mg/g d.m.) obtained after 3 min of treatment (16.2 kJ; APD 0.648 kJ/mL), followed by a significant decrease (0.476 mg/g d.m.) after 5 min. This suggests that moderate ultrasound exposure may enhance pigment release, whereas prolonged treatment may negatively affect pigment stability. Extended cavitation generates structural shear stress and localised microheating around collapsing bubbles, which can accelerate the structural breakdown of sensitive macromolecular complexes and target pigments. The observed variations in chlorophyll content are in agreement with the findings of Ebrahimi et al. (2025), who demonstrated that chlorophyll recovery from sugar beet leaves during UAE strongly depends on extraction conditions, particularly solvent composition and processing parameters [2]. Their study showed that ultrasound processing conditions can be optimised either to minimise chlorophyll co-extraction or to enhance pigment recovery, depending on the intended application. In the present study, the highest chlorophyll content was obtained after 3 min of sonication in methanolic extracts, while prolonged treatment resulted in a decrease, indicating that pigment recovery is not solely dependent on treatment duration. Overall, these findings further confirm that chlorophyll extraction by ultrasound is highly dependent on the selected extraction conditions and should therefore be optimised according to the desired extraction outcome.

### 3.3. Effects of Microwave-Assisted Extraction (MAE)

**Figure 4:**
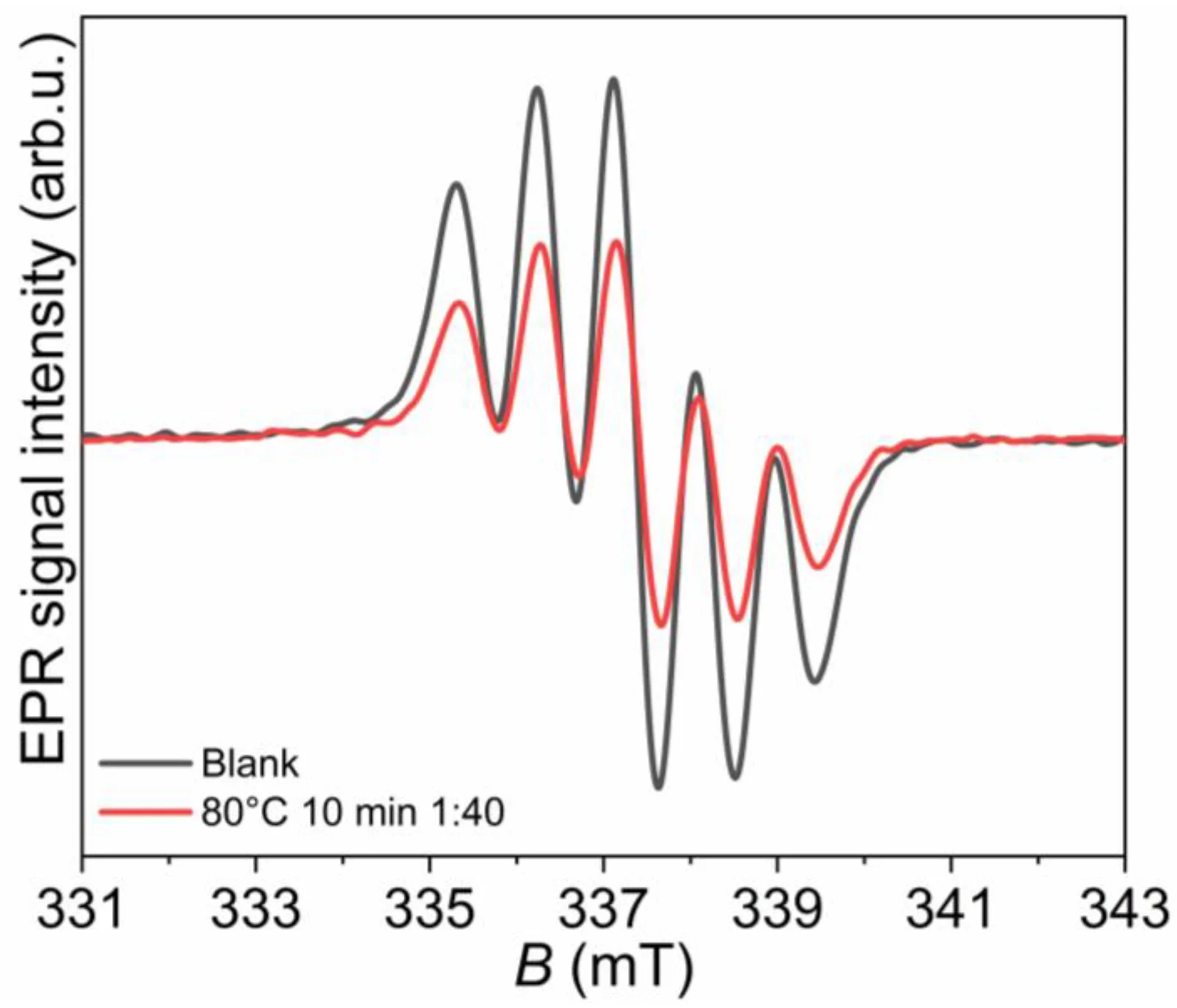
Comparison of EPR spectra of the blank sample and the highest AA achieved at 80 °C for 10 min under an SSR of 1:40.

The results from this study confirm that MAE is an effective technique for the recovery of bioactive compounds from SBL, although the optimal processing conditions remain highly dependent on the targeted bioactive compounds. The observed differences across the evaluated parameters can be fundamentally explained by the mechanism of MAE, in which rapid volumetric heating promotes cell disruption and enhances mass transfer, facilitating the efficient release of intracellular compounds into the extraction solvent [26]. This targeted thermal action overcomes matrix resistance more effectively than non-thermal technologies. However, the distinct responses of individual bioactive compounds depend on their unique thermal stability, structural localisation, and extraction behaviour, which explains why different optimal conditions were observed for TPC, chlorophylls, and AA. The results indicate that the effect of the SSR on extraction efficiency was heavily dependent on the target bioactive compounds. While higher solvent availability generally favoured the recovery of polyphenols and AA, a lower SSR promoted chlorophyll recovery. Similar observations have been reported for MAE, where the SSR plays an important role in balancing microwave energy absorption, matrix heating, and mass transfer processes [16]. In the case of polyphenols, a larger solvent volume (e.g., 1:40 SSR) combined with moderate thermal energy at 60 °C provided an adequate concentration gradient for effective dissolution and mass transfer, yielding the highest nominal TPC values ((23.75 ± 2.32) mg GAE/g d.m.). In contrast, increasing temperature generally enhances diffusion and solubilisation of target compounds, although excessive thermal input may promote the degradation of thermolabile constituents [27,28]. This is particularly evident under a restricted solvent volume (1:20 SSR) at 80 °C, where TPC dropped to its absolute minimum of (2.90 ± 1.58) mg GAE/g d.m., suggesting localised overheating and subsequent thermal degradation of sensitive phenolic structures. Chlorophylls exhibited a similarly high sensitivity to thermal conditions, showing a clear preference for a lower extraction temperature (60 °C), with maximum pigment retention achieved at 60 °C for 10 min under a 1:20 SSR ((0.029 ± 0.000) mg/g d.m.). Elevated temperatures (80 °C) across almost all configurations accelerated pigment degradation due to the thermal cleavage of the chlorophyll molecules. Interestingly, the highest AA ((44.57 ± 4.47) mg AAE/g d.m.) was achieved at 80 °C for 10 min under an SSR of 1:40 (Figure 4).

The particularly low AA values observed under certain extraction conditions indicate a limited reduction in the EPR-DPPH signal compared with the control. As AA was calculated from the calibration curve, small differences in signal intensity may translate into low calculated AA values, particularly when the response is close to that of the control. These results should therefore be interpreted in the context of the overall variability across extraction conditions. In the end, these findings suggest that the antioxidant activity of SBL extracts was not solely associated with TPC but also reflected the contribution of other bioactive constituents extracted under these specific processing conditions, or the formation of novel, heat-induced antioxidant structures. Therefore, the optimal extraction conditions, among the investigated, for MAE must be selected according to the desired extraction outcome. Importantly, these findings are in agreement with those reported by Maravić et al. (2022), who identified MAE as one of the most efficient techniques for recovering polyphenols from sugar beet leaves, outperforming conventional extraction methods in terms of TPC and AA [6]. Likewise, Talebi Haghgo et al. (2026) demonstrated that high extraction efficiencies can be achieved under optimized MAE conditions [29]. In agreement with these studies, the present results confirm that while MAE is a robust tool for valorising SBL biomass, parameters must be meticulously adjusted based on compound-specific traits.

### 3.4. Comparative Performance of Extraction Technologies

**Figure 5:**
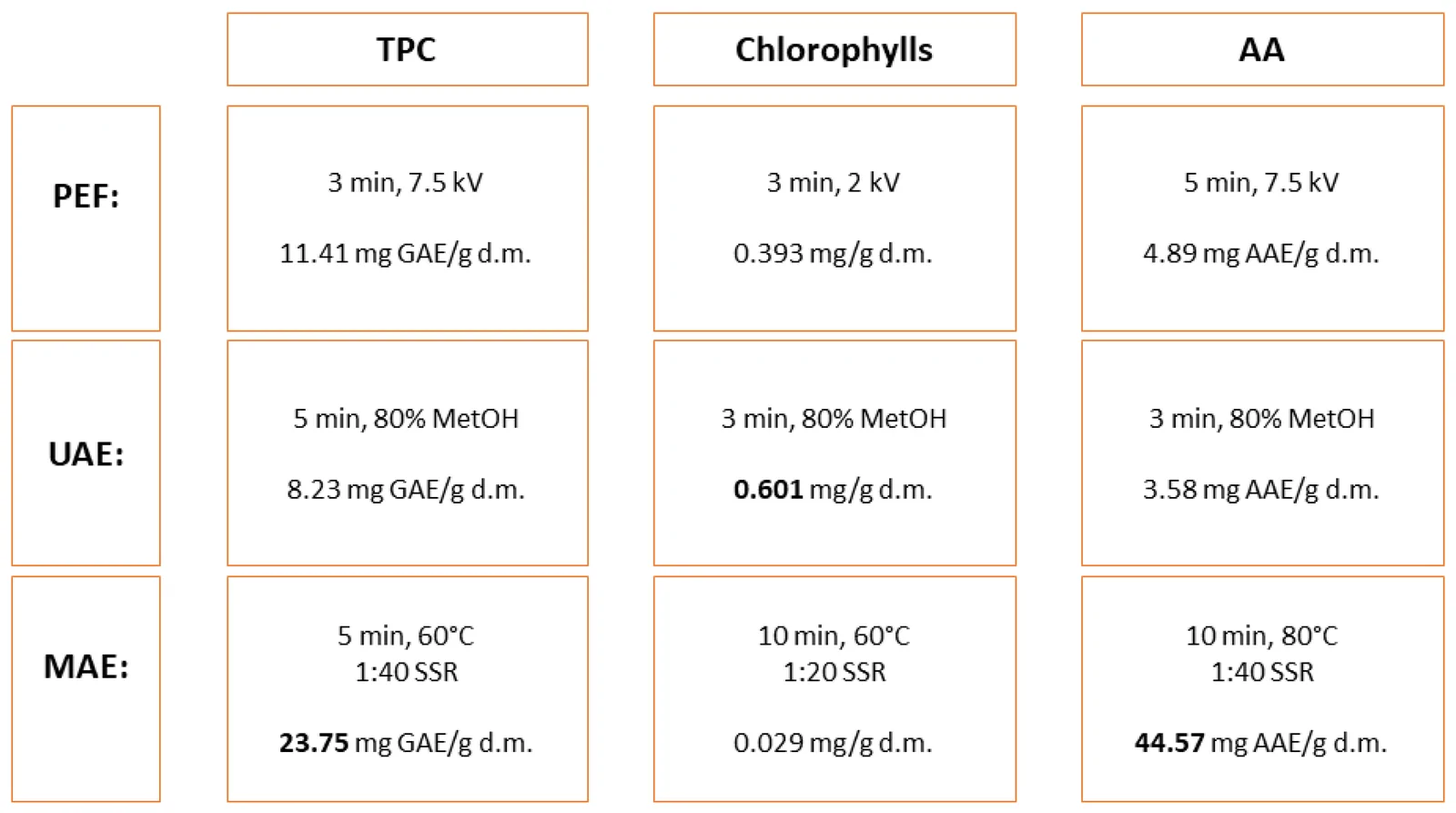
Processing conditions yielding the highest measured values of TPC, chlorophyll content, and AA for each extraction technology (PEF, UAE, and MAE) within the investigated experimental ranges. Bold numbers indicate the highest values of each parameter among the three extraction technologies.

A comprehensive comparison of PEF, UAE, and MAE reveals distinct differences in their efficiency when applied to the SBL matrix as shown with the hierarchical clustering approach (Figure 3). The conditions yielding the highest measured values varied according to both the target compounds and the extraction technology. Figure 5 summarises the processing conditions associated with the highest measured values of TPC, chlorophyll content, and AA in SBL extracts obtained using PEF, UAE, and MAE.

According to the presenting results, MAE resulted in the highest TPC and AA, while the same conditions yielded the lowest chlorophyll content among the investigated technologies. In contrast, UAE provided the highest chlorophyll recovery but the lowest TPC and AA among the investigated technologies. PEF exhibited intermediate performance for all three response variables. It should be noted that ethanol was selected for MAE as a more environmentally favourable solvent, particularly given the larger solvent volumes required. However, the use of different solvents across extraction techniques should be considered when comparing their performance, as the observed differences may be influenced by both the extraction method and solvent properties. Furthermore, these comparisons should be interpreted with caution, as the identified optimal conditions represent the best-performing conditions within the investigated experimental ranges and do not necessarily reflect the true optimum for each extraction technology.

Despite the differences in the conditions associated with the highest measured values among the investigated response variables, each extraction technology exhibited characteristic trends. In PEF, a treatment time of 3 min was associated with the highest TPC, chlorophyll content, and AA, whereas the electric field strength associated with the highest measured values varied according to the target compound. Similarly, in UAE, 80% methanol was consistently identified as the optimal extraction solvent, while the optimal extraction time varied only slightly between 3 and 5 min. In MAE, the processing conditions associated with the highest measured values varied depending on the targeted response. These observations indicate that extraction efficiency depends on the balance between cell disruption, solvent penetration, mass transfer, and preservation of individual bioactive compounds, rather than simply increasing treatment intensity.

The differences between these results reflect the distinct extraction mechanisms governing each technology, including electroporation in PEF, acoustic cavitation during ultrasonication, and dielectric heating during MAE, as well as the influence of processing variables such as treatment intensity, temperature, solvent properties, particle size, and SSR [30].

In PEF treated samples, the highest TPC and AA were obtained at the highest applied electric field strength (7.5 kV/cm), whereas the highest chlorophyll recovery was achieved at a lower field strength (2 kV/cm). This observation suggests that the intensity of the applied electric field affected the extraction of different classes of compounds to different extents. PEF promotes the formation of pores in cell membranes through electroporation, thereby enhancing cell permeability and facilitating the diffusion of intracellular compounds into the extraction solvent [31,32]. However, the different electric field strengths associated with the highest measured values for phenolic compounds and chlorophylls suggest that membrane permeabilisation alone cannot fully explain extraction efficiency. Instead, the physicochemical properties and intracellular localisation of individual compounds likely determine their release under different electroporation conditions [33]. These findings indicate that optimisation of PEF extraction should be tailored to the target compounds, as processing conditions that maximise the recovery of phenolic compounds may not be equally effective for chlorophyll extraction due to the rigid structural resistance of the waxy cuticle and the cell wall.

In the present study, UAE resulted in the highest chlorophyll concentration among the investigated extraction technologies. However, this represented a single treatment yielding the highest measured value, whereas PEF-treated samples generally exhibited higher chlorophyll contents across most of the investigated processing conditions, indicating a more consistent extraction performance. The higher chlorophyll recovery achieved under the ultrasound conditions associated with the highest measured value may be attributed to acoustic cavitation, where the formation and collapse of microbubbles disrupt plant cell structures, enhance solvent penetration, and facilitate mass transfer, thereby promoting the release of intracellular pigments into the extraction solvent [34,35,36]. Compared with MAE, the relatively mild thermal conditions employed during UAE may contribute to the preservation of thermolabile pigments such as chlorophylls by limiting their thermal degradation. Furthermore, acoustic cavitation enhances solvent penetration into plant tissues without extensive heating, allowing efficient pigment release while preserving chlorophyll integrity. This may explain why UAE outperformed both PEF and MAE. Additionally, the identification of 80% methanol as the most effective extraction solvent suggests that solvent composition also played an important role in chlorophyll recovery by providing favourable conditions for pigment solubilisation and mass transfer during ultrasonication.

The superior extraction efficiency of MAE observed in the present study for phenolic and antioxidant components can be attributed to rapid volumetric heating, which enhances solvent penetration, increases mass transfer, and promotes the release of intracellular compounds [25,26]. However, the microwave conditions associated with the highest measured values depended on the target response variable. In agreement with these findings, the highest TPC was obtained at 60 °C for 5 min using an SSR of 1:40, whereas the highest AA was achieved at 80 °C for 10 min and an SSR of 1:40. These findings suggest that the AA of SBL extracts was not solely determined by TPC, but also reflected the contribution of other bioactive constituents extracted under different microwave conditions. Molecules with a permanent dipole moment, such as phenolic compounds, and ionic solutions strongly absorb microwave energy. Microwaves also induce internal superheating of water molecules, promoting cell disruption and enhancing the recovery of target compounds. In addition, the generated thermal energy improves the solubility and diffusion of phenolic compounds and enhances mass transfer [37], which may partially explain the higher TPC achieved by MAE compared with PEF and UAE under the investigated conditions. However, this distinct advantage of MAE was not observed for chlorophyll recovery. Both PEF and UAE resulted in considerably higher chlorophyll contents than MAE, confirming that chlorophyll extraction is governed by different mechanisms than phenolic extraction. The superior pigment recovery obtained by the non-thermal technologies (PEF and UAE) is likely associated with reduced thermal exposure, which helps preserve thermolabile chlorophyll molecules from structural cleavage and pheophytinisation. Ultimately, as confirmed by Maravić et al. (2022) and Talebi Haghgo et al. (2026), while MAE provides unprecedented power for volumetric disruption and mass transfer of robust matrix components [6,29], parameters must be meticulously balanced against compound-specific traits to prevent the collateral degradation of delicate target bioactives.

These findings demonstrate that no single extraction technology was equally effective for maximising all investigated responses, highlighting the importance of selecting extraction conditions according to the targeted application profile of the final product. However, the observed differences reflect the combined effects of the extraction technologies, solvent compositions, and processing conditions investigated. Therefore, the results allow a comparison of the outcomes obtained under the specific experimental conditions used in this study, but do not establish the intrinsic superiority of one extraction technology over another independently of solvent effects and other processing variables.

## 4. Materials and Methods

### 4.1. Sugar Beet Leaves (SBL) Sample Preparation

Sugar beet leaves (SBL; *Beta vulgaris* var. *saccharifera* L.) were obtained from the Croatian family business “Hrgović” in Kapinci, Croatia (45.8126° N, 17.7008° E). The SBL samples were dried in a STATUS fruit, vegetable and herb dehydrator (Status d.o.o., Metlika, Slovenia) at 50 °C to a constant mass, ground in a mill (Bosch TSM6A011W, BSH Hausgeräte GmbH, Munich, Germany) to a fine powder, and stored in plastic cups at room temperature until further analysis.

### 4.2. Processing Conditions

#### 4.2.1. Pulsed Electric Field (PEF) Treatments

The PEF reactor (HVG60/1, Impel Ltd., Zagreb, Croatia) consisted of two parallel stainless-steel electrodes: a high-voltage electrode and a grounded electrode, each with a diameter of 68 mm, separated by a distance of 1 cm. A total of 2 g of SBL powder was suspended in 25 mL of an 80% methanol solution (VWR, Vienna, Austria) corresponding to the working volume of the PEF reactor and providing sufficient solvent for sample suspension during treatment. The samples were subjected to PEF treatment according to the experimental design, where treatment duration (0–5 min) and applied voltage (0–7.5 kV) at a 2 μs pulse duration and a pulse frequency of 150 Hz were used as independent variables to evaluate the effects of these two parameters without simultaneously varying pulse duration. Considering the 1 cm electrode gap, the corresponding applied electric field strength ranged from 0 to 7.5 kV/cm. The total number of electrical pulses applied during the PEF treatment was calculated according to the following equation:

(1) N=f·t 

where N is the total number of pulses, f is the pulse frequency (Hz), and t is the treatment duration (s).

The total effective pulse-on time was calculated from the pulse duration using the following equation:

(2) $$t_{ON}=N\;\cdot \;t_{p}$$

where $t_{ON}$ is the cumulative pulse-on time (s), N is the total number of pulses, and $t_{p}$ is the duration of an individual pulse (2 μs). The values of total effective pulse-on time and individual pulse duration are presented in Table 5.

After PEF treatment, the samples were centrifuged with a Thermo Scientific Heraeus Multifuge 3S-R Refrigerated Centrifuge (Waltham, MA, USA) at 7000 rpm for 10 min. For the control sample (PEF-untreated), the isolation of total polyphenolic compounds (TPC) and chlorophylls was carried out using the conventional solvent extraction method. After adding the solvent, the control sample was vortexed (V-1 Plus, Biosan, Riga, Latvia) for 10 min and subsequently centrifuged at 7000 rpm for 10 min.

#### 4.2.2. Ultrasound-Assisted Extraction (UAE) Treatments

Ultrasound treatments were performed using a 27.8 kHz ultrasound generator (UWave 2000 Multipurpose Microwave Chemistry Workstation; Shanghai Sineo Microwave Chemistry Technology Co., Ltd., Shanghai, China) equipped with a 14 mm diameter probe. Depending on the solvent used (25 mL of 96% ethanol (Gram-mol, Zagreb, Croatia) or 80% methanol), 2 g of SBL was suspended and ultrasonicated for 0, 1, 3, or 5 min at 40% amplitude. Methanol and ethanol were compared to evaluate their extraction performance and assess the potential of ethanol as an alternative solvent. An ultrasound amplitude of 40% was selected based on preliminary trials: at 30%, extraction was insufficient, whereas at 50%, excessive heating was observed, potentially increasing the risk of thermal degradation of the extracted compounds. During the ultrasound treatments, the sample temperature did not exceed 40 °C. Following ultrasonication, the samples were centrifuged at 7000 rpm for 10 min. Control samples (without ultrasound treatment) were extracted under identical conditions, omitting the ultrasonication step. The ultrasound treatment parameters (amplitude, duration, current, acoustic power, total energy, and acoustic power density) are summarised in Table 6. The total energy input during ultrasonication was calculated according to the following equation:

(3) E=P·t

where E is the total energy input (kJ), P is the acoustic power (W), and t is the treatment duration (s).

The acoustic power density (APD,kJ/mL) was calculated as:

(4) $$APD=\frac{E}{V}$$

where E is the total energy input (kJ), and V is the volume of the extraction medium (mL).

#### 4.2.3. Microwave-Assisted Extraction (MAE) Treatments

The experimental procedure was based on a previously published study employing the same microwave extraction system, although a different plant matrix (chokeberry pomace) was investigated in that study [38]. Samples were treated using an Ethos Easy reactor (Milestone, Sorisole, Italy), a MAE system. The required amount of SBL powder was weighed into the extraction vessel according to the selected solvent-to-solid ratio (SSR). Subsequently, 40 mL of 30% ethanol and a magnetic stir bar were added. The vessel containing the sample mixture was placed in the reactor, and extraction was carried out according to the experimental design (Table 7), with temperature, extraction time, and SSR as independent variables. Microwave power (400 W), preheating time (3 min), post-extraction aeration/cooling time (1 min) and stirring intensity (50%) were kept constant throughout all experiments.

### 4.3. Bioactive Compound Content, Pigment Profile, and AA

#### 4.3.1. Determination of Total Polyphenolic Compounds

After each treatment, the samples were centrifuged, and the supernatants were separated from the precipitates. TPC was determined in the supernatants using the Folin–Ciocalteu (Merck, Kenilworth, NJ, USA) assay as previously described by Pleslić et al. [10]. Absorbance was measured at 765 nm using a Secomam UviLine 9400 spectrophotometer (Secomam Groupe Aqualabo, Alès, France). A gallic acid (Thermo Scientific^TM^ Chemicals, Waltham, MA, USA) calibration curve (0.06–0.30 mg/mL) was used to calculate the concentration of polyphenolic compounds extracted from SBL. The results are expressed as milligrams of gallic acid equivalents (GAE) per gram of dry matter (mg GAE/g d.m.).

#### 4.3.2. Determination of Total Chlorophylls

After decanting the supernatant, 25 mL of an 80% acetone (Kemika, Zagreb, Croatia) solution was added to the precipitate for chlorophyll extraction. The resulting suspension was vortexed for 10 min and subsequently centrifuged at 2500 rpm for 10 min. Absorbance was measured at 665 and 645 nm using a Secomam UviLine 9400 spectrophotometer (Secomam Groupe Aqualabo, Alès, France). The total chlorophyll concentration in the extract (was calculated according to the equation of Vayupharp & Laksanalamai (2013) [39]:

(5) $$total\;chlorophyll\;content=7.15\;A_{665}+18.71\;A_{645}$$

where $A_{665}$ and $A_{645}$ represent the absorbances measured at 665 and 645 nm, respectively. The results are expressed as mg of total chlorophylls per g of dry matter (mg/g d.m.).

#### 4.3.3. Determination of AA

Electron paramagnetic resonance (EPR) spectroscopy combined with the DPPH assay was employed to determine the antioxidant activity of SBL samples, following the protocol described by Pleslić et al. [10]. Measurements were performed using an X-band EPR spectrometer (MiniScope MS 5000, Magnettech, Berlin, Germany). A DPPH solution was prepared by dissolving DPPH (Sigma, St. Louis, MO, USA) in 96% ethanol to a final concentration of 15 mM. A volume of 970 μL of DPPH solution was mixed with 30 μL of sample, after which EPR spectra were recorded for 30 min. The EPR experimental parameters were set as follows: magnetic field sweep from 331 to 343 mT, modulation amplitude of 0.2 mT, modulation frequency of 100 kHz, and microwave power of 10 mW. The antioxidant activity of the samples is expressed as milligrams of ascorbic acid equivalents (AAE) per gram of dry matter (mg AAE/g d.m.) using an L(+)-ascorbic acid (Gram-mol, Zagreb, Croatia) calibration curve (0.01–0.4 mg/mL).

#### 4.3.4. Statistical Analysis

All measurements were performed in triplicate, and the results are presented as mean ± standard deviation (SD). Statistical analyses were conducted using Systat v.13.2.01 (Grafiti LLC, Palo Alto, CA, USA). The effects of the three extraction technologies and their processing parameters on the measured responses were evaluated using one-way and factorial analysis of variance (ANOVA), depending on the experimental design. When applicable, differences between individual group means were assessed using Tukey’s post hoc test, and statistical significance was set at *p* ≤ 0.05.

To evaluate the overall extraction efficiency and elucidate the natural groupings among the different advanced extraction technologies, a Hierarchical Cluster Analysis (HCA) coupled with a Clustered Heatmap visualisation was performed. Clustering was based entirely on three key parameters: TPC, chlorophyll content, and AA. To eliminate any scaling bias, the raw mean values were normalised using standard Z-score transformation. The final tree was then calculated applying Ward’s agglomerative minimum variance method and Euclidean distance metrics.

## 5. Conclusions

The present study provides a comprehensive comparative evaluation of three emerging green extraction technologies—pulsed electric field (PEF), ultrasound-assisted extraction (UAE), and microwave-assisted extraction (MAE)—for the recovery of total polyphenols, chlorophylls, and antioxidant compounds from sugar beet leaves (SBL). The experimental findings clearly demonstrate that no single extraction technology is equally effective for maximising all target variables simultaneously, as extraction efficiency is strongly governed by the specific interaction between the technological mechanism and the physicochemical properties of the bioactive compounds. Each extraction technology demonstrated specific advantages depending on the targeted bioactive compounds.

MAE showed the highest efficiency for the recovery of phenolic compounds and antioxidant activity, achieving the highest TPC (23.75 mg GAE/g d.m.) and AA (44.57 mg AAE/g d.m.) among the investigated approaches. This pathway is driven by rapid volumetric heating and intense cellular disruption, although it triggers substantial degradation of thermosensitive pigments at higher temperatures (80 °C) when paired with lower solvent ratios (1:20 SSR).

UAE provided the highest chlorophyll recovery (0.601 mg/g d.m.), highlighting its suitability for pigment preservation under mild processing conditions. Within this approach, solvent chemistry represented the dominant factor, with 80% methanol significantly outperforming 96% ethanol, while extended sonication (> 3 min) induced localised degradation trends.

PEF extraction resulted in a moderate numerical improvement compared with the other extraction approaches. However, the numerical trends varied depending on the target compound and processing conditions, indicating that the response to PEF treatment was not uniform across the measured variables.

The results obtained in the present study should primarily be considered as a screening-level assessment of whether the investigated extraction technologies enhance or reduce the overall recovery of TPC, chlorophylls and AA. It should be noted that the best-performing conditions identified in this study represent the most favourable conditions among those investigated and do not necessarily correspond to the true optimum for the extraction of bioactive compounds from sugar beet leaves. Further investigation of a broader range of experimental conditions would be required to establish the optimum extraction parameters for this matrix.

This study highlights the importance of technology-specific extraction strategies for maximising the recovery of valuable compounds from this underutilised plant material. Furthermore, these observations indicate that extraction efficiency depends on a compound-dependent balance between cell disruption, solvent penetration, mass transfer, and the preservation of individual bioactive structures, rather than simply increasing treatment intensity. These findings provide a valuable basis for selecting appropriate extraction technologies depending on the intended application of SBL extracts, whether for food, feed, or nutraceutical purposes.

Further research should focus on expanding the investigated processing parameters to achieve greater extraction efficiency and better understand the response of sugar beet leaves to different extraction mechanisms. Furthermore, future studies should also include HPLC-based characterization of the individual phenolic compounds in extracts obtained under the optimal processing conditions identified for each extraction technology, providing a more detailed assessment of how PEF, UAE, and MAE affect the phenolic profile of sugar beet leaves.

Optimization of ultrasound intensity, PEF treatment parameters, and microwave conditions, as well as the application of combined or sequential extraction approaches, could provide additional opportunities to enhance the recovery of bioactive compounds while preserving sensitive constituents.

## Figures and Tables

**Table 1:** Analysis of variance (ANOVA) results for total phenolic content (TPC), chlorophylls, and antioxidant activity (AA) of SBL extracts.

Dependent Variable	Source of Variation	Sum of Squares	*df*	Mean Square	*F*-Value	*p*-Value
TPC	Between groups	13.4113	9	1.49015	1.70	0.2090
	Within groups	8.74435	10	0.87444		
	Total	22.1557	19			
Chlorophylls	Between groups	0.04137	9	0.00460	7.92	0.0017 **
	Within groups	0.00581	10	0.00058		
	Total	0.04718	19			
AA	Between groups	0.01192	9	0.00132	2.24	0.1119
	Within groups	0.0059	10	0.00059		
	Total	0.01782	19			

** Statistically significant at p ≤ 0.01.

**Table 2:** ANOVA results for the effects of ultrasound treatment parameters (duration and solvent type) on TPC, chlorophyll content, and antioxidant activity of SBL extracts.

Dependent Variable	Source of Variation/Interactions	*df*	*F*-Value	*p*-Value
TPC	Treatment duration	3	2.305	0.153
	Solvent type	1	594.264	<0.001 *
	Treatment duration × Solvent type	3	0.686	0.585
Chlorophylls	Treatment duration	3	18.118	<0.001 *
	Solvent type	1	281.617	<0.001 *
	Treatment duration × Solvent type	3	17.319	<0.001 *
AA	Treatment duration	3	0.307	0.820
	Solvent type	1	46.165	<0.001 *
	Treatment duration × Solvent type	3	0.262	0.850

* Statistically significant at p ≤ 0.01.

**Table 3:** Total polyphenolic content (TPC), chlorophyll content, and antioxidant activity of SBL extracts obtained under different MAE conditions (temperature, treatment duration, and solid-to-solvent ratio (SSR)).

Temperature (°C)	Treatment Duration (min)	SSR (Solid-to-Solvent Ratio)	TPC (mg GAE/g d.m.)	Chlorophylls (mg/g d.m.)	AA (mg AAE/g d.m.)
60	5	1:20	17.26 ± 0.64 **^B^**^ay^	0.027 ± 0.002 **^B^**^ax^	28.51 ± 2.21 **^A^**^az^
		1:30	15.88 ± 0.57 **^A^**^ax^	0.022 ± 0.000 **^B^**^ay^	3.60 ± 1.24 **^A^**^ax^
		1:40	23.75 ± 2.32 **^A^**^ax^	0.014 ± 0.000 **^A^**^ay^	35.04 ± 3.98 **^B^**^by^
	10	1:20	23.38 ± 2.44 **^A^**^ay^	0.029 ± 0.000 **^B^**^az^	37.72 ± 1.96 **^A^**^ax^
		1:30	17.11 ± 1.52 **^A^**^ax^	0.022 ± 0.001 **^B^**^ay^	23.37 ± 1.72 **^A^**^ax^
		1:40	16.03 ± 1.61 **^A^**^ax^	0.017 ± 0.001 **^A^**^bx^	31.50 ± 2.86 **^A^**^ax^
80	5	1:20	2.90 ± 1.58 **^A^**^ax^	0.017 ± 0.001 **^A^**^ay^	30.21 ± 1.24 **^A^**^ax^
		1:30	19.52 ± 4.93 **^A^**^az^	0.018 ± 0.002 **^A^**^ay^	17.47 ± 1.68 **^B^**^ax^
		1:40	18.80 ± 1.37 **^A^**^axz^	0.013 ± 0.001 **^A^**^ax^	<0.01 ± 1.38 **^A^**^ax^
	10	1:20	15.32 ± 1.16 **^A^**^bx^	0.018 ± 0.001 **^A^**^az^	10.50 ± 1.13 **^A^**^ax^
		1:30	17.29 ± 4.11 **^A^**^ax^	0.016 ± 0.000 **^A^**^axz^	31.99 ± 0.03 **^A^**^by^
		1:40	19.64 ± 1.51 **^A^**^ax^	0.014 ± 0.001 **^A^**^ax^	44.57 ± 4.47 **^B^**^bz^

*Within each column, different uppercase letters (A, B) indicate statistically significant differences between temperature and different lowercase letters (a, b) indicate statistically significant differences between treatment duration and different lowercase letters (x, y, z) indicate statistically significant differences between SSR (p ≤ 0.05, Tukey post hoc test).*

**Table 4:** ANOVA results for the effects of microwave treatment parameters on TPC, chlorophyll content, and antioxidant activity of SBL extracts.

Dependent Variable	Source of Variation/Interactions	*df*	*F*-Value	*p*-Value
TPC	Temperature (Temp)	1	27.869	<0.001 **
	Treatment duration	1	13.739	0.003 **
	SSR	2	19.717	<0.001 **
	Temp × Treatment duration	1	4.518	0.055
	Temp × SSR	2	36.521	<0.001 **
	Treatment duration × SSR	2	28.387	<0.001 **
	Temp × Treatment duration × SSR	2	4.012	0.046 *
Chlorophylls	Temperature (Temp)	1	251.51	<0.001 **
	Treatment duration	1	4.210	0.063
	SSR	2	177.81	<0.001 **
	Temp × Treatment duration	1	6.710	0.023 *
	Temp × SSR	2	42.970	<0.001 **
	Treatment duration × SSR	2	5.100	0.025 *
	Temp × Treatment duration × SSR	2	0.240	0.788
AA	Temperature (Temp)	1	1.426	0.255
	Treatment duration	1	1.426	0.255
	SSR	2	3.290	0.095
	Temp × Treatment duration	1	8.869	0.004 *
	Temp × SSR	2	0.209	0.656
	Treatment duration × SSR	2	3.169	0.051
	Temp × Treatment duration × SSR	2	6.393	0.013 *

* Statistically significant at p ≤ 0.05, ** statistically significant at p ≤ 0.01.

**Table 5:** Processing parameters for PEF treatments of SBL samples.

Treatment Duration (min)	Number of Pulses	Total Effective Pulse-on Time (s)	Voltage (kV)
0	0	0	0
1	9000	0.018	2
1	9000	0.018	5
1	9000	0.018	7.5
3	27,000	0.054	2
3	27,000	0.054	5
3	27,000	0.054	7.5
5	45,000	0.09	2
5	45,000	0.09	5
5	45,000	0.09	7.5

**Table 6:** Ultrasound treatment parameters applied during the extraction process.

Amplitude (%)	Duration (min)	Current (A)	Acoustic Power (W)	Total Energy (kJ)	Acoustic Power Density, APD (kJ/mL)
40	1	0.30	90	5.4	0.216
	3	0.30	90	16.2	0.648
	5	0.30	90	27.0	1.080

**Table 7:** Experimental design for MAE of SBL.

Temperature (°C)	Treatment Duration (min)	SSR
60	5	1:20
60	5	1:30
60	5	1:40
60	10	1:20
60	10	1:30
60	10	1:40
80	5	1:20
80	5	1:30
80	5	1:40
80	10	1:20
80	10	1:30
80	10	1:40

## Data Availability

The datasets generated during the current study are available from the corresponding authors.

## References

[1] Antczak-Chrobot A., Macierzynski J., Wojtczak M. (2026). Polyphenols in Sugar Beet Leaves: Composition, Variability, and Valorization Opportunities. Molecules.

[2] Ebrahimi P., Bayram I., Mihaylova D., Lante A. (2025). A Strategy to Minimize the Chlorophyll Content in the Phenolic Extract of Sugar Beet Leaves: Can this Extract Work as a Natural Antioxidant in Vegetable Oils?. Food Bioprocess Technol..

[3] Wani F.A., Rashid R., Jabeen A., Brochier B., Yadav S., Aijaz T., Makroo H.A., Dar B.N. (2021). Valorisation of food wastes to produce natural pigments using non-thermal novel extraction methods: A review. Int. J. Food Sci. Technol..

[4] Zia S., Khan M.R., Shabbir M.A., Maan A.A., Khan M.K.I., Nadeem M., Khalil A.A., Din A., Aadil R.M. (2022). An Inclusive Overview of Advanced Thermal and Nonthermal Extraction Techniques for Bioactive Compounds in Food and Food-related Matrices. Food Rev. Int..

[5] Ebrahimi P., Mihaylova D., Marangon C.M., Grigoletto L., Lante A. (2022). Impact of Sample Pretreatment and Extraction Methods on the Bioactive Compounds of Sugar Beet (*Beta vulgaris* L.) Leaves. Molecules.

[6] Maravić N., Teslić N., Nikolić D., Dimić I., Šereš Z., Pavlić B. (2022). From agricultural waste to antioxidant-rich extracts: Green techniques in extraction of polyphenols from sugar beet leaves. Sustain. Chem. Pharm..

[7] Lante A., Ebrahimi P., Mihaylova D. (2022). Comparison of green technologies for valorizing sugar beet (*Beta vulgaris* L.) leaves. Food Sci. Appl. Biotechnol..

[8] Goktayoglu E., Oztop M.H., Ozcan S. (2023). Proteomics Approach to Differentiate Protein Extraction Methods in Sugar Beet Leaves. J. Agric. Food Chem..

[9] Semencic M.C., Dukic J., Slavica A., Saric B., Kubasek M., Jambrak A.R. (2025). Valorization of sugar beet leaves: Optimizing ultrasound-assisted extraction of bioactive substances and proteins. CyTA-J. Food.

[10] Pleslić S., Markić F., Vukušić Pavičić T., Stulić V., Maltar-Strmečki N. (2025). Plasma-Assisted Extraction of Bioactive Compounds from Tomato Peels and Sugar Beet Leaves Monitored by Electron Paramagnetic Resonance Spectroscopy. Appl. Sci..

[11] Narzary K., Brahma R., Boruah B., Rajbongshi M., Ray S. (2026). Comparative Assessment of Thermal and Non-Thermal Processing of Green Leafy Vegetables: A Comprehensive Review. Food Rev. Int..

[12] Zin M.M., Anucha C.B., Banvölgyi S. (2020). Recovery of Phytochemicals via Electromagnetic Irradiation (Microwave-Assisted-Extraction): Betalain and Phenolic Compounds in Perspective. Foods.

[13] Arjeh E., Khodaei S.M., Barzegar M., Pirsa S., Sani I.K., Rahati S., Mohammadi F. (2022). Phenolic compounds of sugar beet (*Beta vulgaris* L.): Separation method, chemical characterization, and biological properties. Food Sci. Nutr..

[14] Barba F.J., Roohinejad S., Ishikawa K., Leong S.Y., Bekhit A.E.A., Saraiva J.A., Lebovka N. (2020). Electron spin resonance as a tool to monitor the influence of novel processing technologies on food properties. Trends Food Sci. Technol..

[15] Markić F., Muratović S., Pleslić S., Maltar-Strmečki N. (2026). Monitoring free radical generation and antioxidant activity in tomato pulp using electron paramagnetic resonance (EPR): Effects of ultrasound-assisted bioactive extraction. LWT.

[16] Nonglait D.L., Gokhale J.S. (2024). Review Insights on the Demand for Natural Pigments and Their Recovery by Emerging Microwave-Assisted Extraction (MAE). Food Bioprocess Technol..

[17] Nutter J., Fernandez M.V., Jagus R.J., Aguero M.V. (2021). Development of an aqueous ultrasound-assisted extraction process of bioactive compounds from beet leaves: A proposal for reducing losses and increasing biomass utilization. J. Sci. Food Agric..

[18] Yeasmen N., Bhuiyan M.H.R., Gariepy Y., Taherian A.R., Dumont M., Ramaswamy H., Orsat V. (2025). Comparative Evaluation of Ultrasonic, High Pressure, and Pulsed Electric Field Processing on the Extraction and Storage Stability of Betalains From Red Beet By-Products. J. Food Sci..

[19] Tekin I., Akyüz A., Görgüç A., Yilmaz F.M., Ersus S. (2026). Pulsed Electric Field-Assisted Processing of Whole Red Beetroot: A Sustainable Approach to Natural Colorant Production. Food Bioprocess Technol..

[20] Katsimichas A., Stathi S., Dimopoulos G., Giannakourou M., Taoukis P. (2024). Kinetics of pulsed electric fields assisted pigment extraction from Chlorella pyrenoidosa. Innov. Food Sci. Emerg. Technol..

[21] Zhou J.J., Wang M., Berrada H., Zhu Z.Z., Grimi N., Barba F.J. (2022). Pulsed electric fields (PEF), pressurized liquid extraction (PLE) and combined PEF plus PLE process evaluation: Effects on microstructure, biomolecules recovery and Triple TOF-LC-MS-MS polyphenol composition. Innov. Food Sci. Emerg. Technol..

[22] Bocker R., Silva E.K. (2022). Pulsed electric field assisted extraction of natural food pigments and colorings from plant matrices. Food Chem. X.

[23] Aslam R., Alam M.S., Kaur J., Panayampadan A.S., Dar O.I., Kothakota A., Pandiselvam R. (2022). Understanding the effects of ultrasound processng on texture and rheological properties of food. J. Texture Stud..

[24] Taha A., Mehany T., Pandiselvam R., Anusha Siddiqui S., Mir N.A., Malik M.A., Sujayasree O., Alamuru K.C., Khanashyam A.C., Casanova F. (2024). Sonoprocessing: Mechanisms and recent applications of power ultrasound in food. Crit. Rev. Food Sci. Nutr..

[25] Cao S.T., Liang J.C., Chen M.G., Xu C., Wang X.Q., Qiu L., Zhao X.Y., Hu W.X. (2025). Comparative analysis of extraction technologies for plant extracts and absolutes. Front Chem..

[26] López-Salazar H., Camacho-Díaz B.H., Ocampo M.L.A., Jiménez-Aparicio A.R. (2023). Microwave-assisted Extraction of Functional Compounds from Plants: A Review. Bioresources.

[27] Melikoglu M. (2025). Microwave-assisted extraction: Recent advances in optimization, synergistic approaches, and applications for green chemistry. Sustain. Chem. Clim. Action.

[28] Travis C.R., McMaster J., Rivas F. (2026). Advances in Natural Product Extraction: Established and Emerging Technologies. Molecules.

[29] Talebi Haghgo M., Mooraki N., Honarvar M. (2026). Optimization of Microwave-Assisted Extraction of Autumn-Grown Sugar Beet (*Beta vulgaris* L.) Leaf Extract and Evaluation of its Antioxidant Activity. J. Food Sci. Technol..

[30] Usman I., Hussain M., Imran A., Afzaal M., Saeed F., Javed M., Afzal A., Ashfaq I., Al Jbawi E., Saewan S.A. (2022). Traditional and innovative approaches for the extraction of bioactive compounds. Int. J. Food Prop..

[31] Chudasama M., Singh D.K., Pradhan R.C. (2025). Review on Electroporation Mechanisms for PEF-Assisted Extraction and Microbial Inactivation. Food Eng. Rev..

[32] Velusamy M., Rajan A., Radhakrishnan M. (2023). Valorisation of food processing wastes using PEF and its economic advances-recent update. Int. J. Food Sci. Technol..

[33] Ranjha M.M.A.N., Kanwal R., Shafique B., Arshad R.N., Irfan S., Kieliszek M., Kowalczewski P.Ł., Irfan M., Khalid M.Z., Roobab U. (2021). A Critical Review on Pulsed Electric Field: A Novel Technology for the Extraction of Phytoconstituents. Molecules.

[34] Bhargava N., Mor R.S., Kumar K., Sharanagat V.S. (2021). Advances in application of ultrasound in food processing: A review. Ultrason. Sonochem..

[35] Chavan P., Sharma P., Sharma S.R., Mittal T.C., Jaiswal A.K. (2022). Application of High-Intensity Ultrasound to Improve Food Processing Efficiency: A Review. Foods.

[36] Linares G., Rojas M.L. (2022). Ultrasound-Assisted Extraction of Natural Pigments From Food Processing By-Products: A Review. Front. Nutr..

[37] Osorio-Tobón J.F. (2020). Recent advances and comparisons of conventional and alternative extraction techniques of phenolic compounds. J. Food Sci. Technol..

[38] Repajic M., Zoric M., Magnabosca I., Pedisic S., Dragovic-Uzelac V., Garofulic I.E. (2025). Bioactive Power of Black Chokeberry Pomace as Affected by Advanced Extraction Techniques and Cryogrinding. Molecules.

[39] Vayupharp B., Laksanalamai V. (2013). Nutrients and anti-nutrients of high chlorophyll-mungbean sprouts as affected by different periods of germination and sprouting stages. Int. J. Agric. Biol. Eng..

